# The Change in the Composition of *Trichoderma reesei* Carbohydrases Complex as a Result of Gamma Mutagenesis

**DOI:** 10.1177/1178636119848368

**Published:** 2019-05-27

**Authors:** Elena V Kostyleva, Anna S Sereda, Dmitrii O Osipov, Irina A Velikoretskaya, Nina V Tsurikova

**Affiliations:** 1 All-Russian Research Institute of Food Biotechnology—A Branch of FRC of Food, Biotechnology, and Food Safety, Moscow, Russia; 2 Federal Research Centre “Fundamentals of Biotechnology” of the Russian Academy of Sciences, Moscow, Russia

**Keywords:** cellulases, hemicellulases, xylanase, endoglucanase

## Abstract

The filamentous fungus *Trichoderma reesei* is traditionally used as the main industrial producer of cellulases and hemicellulases. Recently, the relevance of carbohydrases hydrolyzing nonstarch polysaccharides of cereals has significantly increased in feed production. In processing of grain raw materials, endodepolymerases, mainly xylanases and endoglucanases, play a key role. Earlier, we carried out gamma mutagenesis of an industrial strain *T. reesei* BCM18.2/KK to increase the proportion of endodepolymerases in its enzyme complex. Endoglucanase activity of the strain was increased 5-fold, while xylanase activity increased more than 8-fold. It was interesting to determine the carbohydrases composition in enzyme preparations obtained from the original and mutant *T. reesei* strains. So, the strains were cultured in laboratory fermenters; concentrated preparations were obtained using freeze dryer. It was established that gamma mutagenesis resulted in significant changes in the carbohydrases complex of the strain. Cellobiohydrolase I being the major carbohydrase in the original strain was absent in the enzyme complex of the mutant. The share of xylanases and endoglucanases in the preparation from the mutant strain increased by 6% and 6.5%, respectively, compared with the preparation from the original strain. The obtained data show the ability of gamma irradiation to affect the component composition of *T. reesei* carbohydrase complex.

For decades, *Trichoderma reesei* strains have been the main source of cellulases and hemicellulases for the industrial production of enzyme preparations (EP). Most of the studies concerning the improvement of *T. reesei* strains were aimed at obtaining EP for the textile, pulp, and paper industry and mainly the production of bioethanol from plant biomass.^1^,^2^ Recently, EP of cellulases and hemicellulases are widely used in fodder production to increase the nutritional value of grain raw materials for monogastric animals and poultry. Cleavage of nonstarch polysaccharides (NSP) in grain by carbohydrases reduces the chyme viscosity, increases the availability of protein and other nutritional components of feeds, normalizes the intestinal microflora, and increases the energy value and digestibility of forages.^3^

The requirements for EP composition and properties depend on the area of their application. In bioethanol production, exodepolymerases, such as cellobiohydrolases (CBHs) and β-glucosidase (BGL), play an important role, ensuring effective saccharification of cellulosic substrates.^2^ In fodder production, the key enzymes are endodepolymerases, mainly, endoglucanases (EG) and xylanases (Xyl), which rapidly reduce the polymerization degree of NSP.^3^

*Trichoderma reesei* produces a complex of cellulases and hemicellulases acting synergistically and providing deep destruction of the most common plant NSP.^1^,^2^ The main components of *T. reesei* cellulase complex are cellobiohydrolase I (CBH I; 59-68 kDa), CBH II (50-58 kDa), EG I (50-55 kDa), and EG II (48 kDa). The share of CBH I is up to 50%-60% of all *T. reesei* cellulases, and the CBH II accounts for about 20%; EG I and EG II comprise about 20% of *T. reesei* cellulases. The minor components are EG III (25 kDa), EG IV (55 kDa), EG V (23 kDa), and β-glucosidase I (BGL I 75 kDa).^1^,^2^,^4^ The content of hemicellulases, represented mainly by Xyl I and II, is approximately 2 times lower than cellulases.^5^ *T. reesei* xylanase activity consists of Xyl I, II, III, IV, and β-xylosidase (β-Xyl, approx. 100 kDa). Major components are Xyl I (19 kDa) and/or Xyl II (21 kDa).^4^,^6^,^7^ So, exodepolymerases CBH I and II amounting about 80% of all cellulases are predominant in *T. reesei* extracellular enzyme complex.^5^

To obtain EP for feeding purposes with an increased proportion of endodepolymerases, we carried out induced mutagenesis of the strain *T. reesei* BCM18.2/KK—an industrial producer of cellulases and hemicellulases (Patent RF 2001949). In general, the expression of cellulases and hemicellulases *T. reesei* is coordinated and regulated by 5 major transcription factors.^8^ While the ratio of cellulases and hemicellulases can be changed by varying the cultivation conditions such as pH, media composition, and so on,^4^ changing the exodepolymerase and endodepolymerases ratio is more difficult task. It is known that random mutagenesis can affect different sites on DNA throughout the genome including coding regions of carbohydrases genes or their promoters.^4^,^9^ So, despite the fact that many of the *T. reesei* genes encoding carbohydrate-active enzymes (CAZymes) are nonrandomly distributed within the genome,^10^ we suggested that γ-rays can affect separately on different genes encoding endo- and exo-carbohydrases. This assumption is supported by the literature data showing that expression pathways of Xyl can be not equally affected by the mutations.^4^

After several stages of gamma irradiation in a GUT-200 chamber with cobalt-60 as radiation source using severe irradiation regimes of 2000 to 2500 Gray and low survival rates of 0.00014% to 0.01%, a *T. reesei*-Co-44 mutant was obtained with a significant increase in EG and Xyl activity.^11^

It was interesting to determine the carbohydrases composition in EP obtained from the initial and mutant *T. reesei* strains. So, the strains were cultured in 3-L laboratory fermenters with a working volume of 1.35 L in a fed-batch mode on a medium of the following composition (%): glucose -5.0, microcrystalline cellulose (MCC) -1, corn extract -4.0, KH_2_PO_4_ -0.2, (NH_4_)_2_SO_4_ -0.6, CaCl_2_ -0.06, antifoam Propinol -0.1, and tap water, pH 4.5. Feeding was carried out in continuous mode with a 30% aqueous solution of lactose and glucose in the ratio of 1:1. After the fermentation, the culture biomass was separated by centrifugation at 6000 r/min for 20 minutes. Concentrated EP were obtained from the culture liquid supernatants using a freeze dryer. In the EP, we determined the soluble protein content (by Lowry method) and the activity of the main carbohydrases by the initial rate of reducing sugars (RS) formation (by Somogyi-Nelson method) using the corresponding substrates: birch xylan for Xyl, barley β-glucan and Na-salt of carboxymethylcellulose (CMC) for EGs, and Avicel for CBHs (Table 1). One unit of activity was defined as an amount of enzyme which is necessary for the formation of 1 µmol RS for 1 minute at 50°C, pH 5.0.

Statistical analysis of the data obtained in no less than 3 replicas was performed using one-way analysis of variance (ANOVA) with STATISTIKA 10 software.

**Table 1.** The specific activity of the main carbohydrases in EP from the original and mutant *Trichoderma reesei* strains.

Characteristic of EP	EP1 (from *T. reesei* BCM 18.2/KK—original)	EP2 (from *T. reesei* Со-44—mutant)	*P* value
Specific activity units/mg of protein			
CMC	8.5 ± 0.41	13.5 ± 0.70	.000434
β-glucan	7.2 ± 0.36	13.1 ± 0.68	.00019
Xylan	2.6 ± 0.13	11.1 ± 0.58	.00016
Avicel	0.37 ± 0.02	0.32 ± 0.015	.02
Soluble protein content (mg/g)	554 ± 28	547 ± 28	.76

Abbreviations: EP, enzyme preparations; CMC, carboxymethylcellulose.

Data are shown as mean ± standard deviation.

In the EP2 from the mutant strain, the specific endo- and β-glucanase activity increased by 1.6 to 1.8 times and the Xyl activity increased by 4.3 times compared with the EP1 from the original strain; the specific activity on Avicel decreased by 15%. It indicates changes toward increasing the endodepolymerases activity.

To detect the changes in content of the major *T. reesei* carbohydrases, sodium dodecyl sulfate-polyacrylamide gel electrophoresis (SDS-PAGE) was performed at EP dosage of 20 μg protein per lane. The SDS-PAGE electrophoresis of EP1 and 2 showed (Figure 1) that significant changes have occurred in the *T. reesei* Co-44 enzyme complex: the 66 to 68 kDa band corresponding to the CBH I disappeared, a band of about 25 kDa corresponding to EG III *T. reesei*, and the bands at 19 to 21 kDa corresponding to Xyl I and II and 48 kDa corresponding to EG II *T. reesei* became more intense.

**Figure 1.**
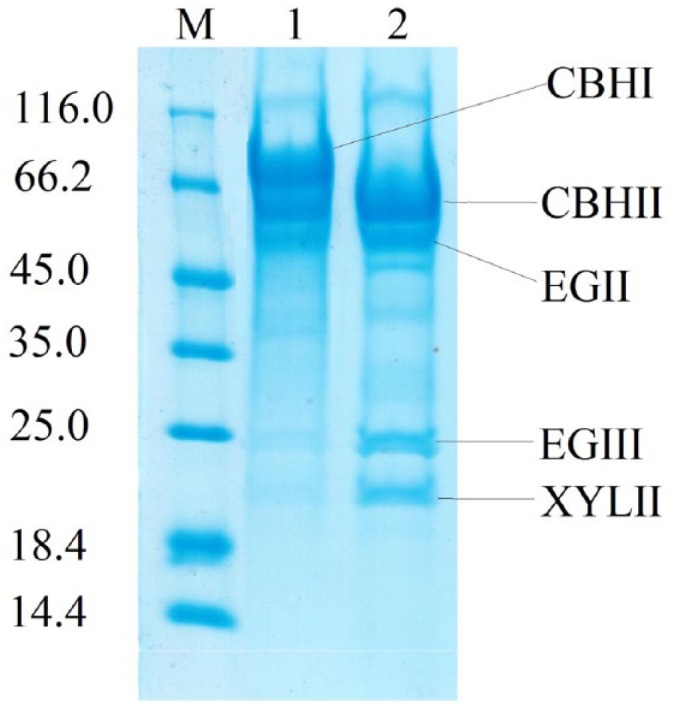
Electrophoresis of enzyme preparations obtained from original strain *Trichoderma reesei*-18.2/KK (1) and the mutant strain *T. reesei*-Со-44 (2). CBH indicates cellobiohydrolases; EG, endoglucanase; XYL, xylanase.

The component composition of EP1 and EP2 was determined by the method of chromatographic protein fractionation, followed by measuring the activities in the obtained fractions with respect to various substrates. The fractionation scheme included 3 stages: desalting of the dissolved EP by gel permeation chromatography; coarse fractionation of the enzyme complex, using anion-exchange chromatography on a Source 15Q carrier; and fine fractionation of protein fractions selected during the previous stage using hydrophobic chromatography on a Source 15 ISO carrier. Activity of carbohydrases was determined in the fractions: CBH—using MCC, EG—using Na- salt of carboxymethyl cellulose, Xyl—using birch xylan, β-xylosidase (bXyl)—using *p*-nitrophenyl-β-D-xylopyranoside, xyloglucanases (XG)—using xyloglucans, β-glucosidase (bGlu)—using *p*-nitrophenyl-β-D-glucoside. The composition of the obtained fractions was characterized by electrophoresis under denaturing conditions in polyacrylamide gel. The protein content in each fraction was determined by absorption at *λ* = 280 nm, and the volume of the fractions was determined by weighing. The fragments of protein bands after SDS-PAGE, corresponding to individual enzymes, were identified by peptide mapping after cleavage of proteins contained in the corresponding gel bands with trypsin (Promega, USA). Matrix-assisted laser desorption/ionization (MALDI) mass spectrometry of trypsin protein hydrolysates was carried out using the time-of-flight mass spectrometer UltrafleXtreme (Bruker Daltonik GmbH, Germany) in the Center for Collective Use “Industrial Biotechnology” of Research Center of Biotechnology, Russian Academy of Sciences. The component composition of the EP1 and EP2 was determined by comparing the obtained electrophoretic, mass spectrometric data and the results of measuring of chromatographic fractions activity toward specific substrates with protein databases (http://www.ncbi.nlm.nih.gov/). To analyze the mass spectrometry results, MASCOT program was used (http://www.matrixscience.com/search_form_select.html). The content of each enzyme in the studied EP was taken as a mass fraction of the enzyme calculated relative to the total amount of protein in the sample and expressed as a percentage.

The fractionation of protein bands of carbohydrases in the EP1 and 2 showed (Figure 2) that CBH1 is indeed completely absent in EP2, and despite a significant increase in the CBH2 proportion (by 20.4%), the total amount of CBHs decreased by 22.4% compared with EP from the original strain. In the EP2 from the mutant strain, the EGs proportion increased by 6.5% and the EG3 content increased to the greatest extent. The share of Xyl in EP2 was 8.5% compared to 2.5% in EP1, and a certain amount of Xyl 3 appeared in EP2, which is absent in the EP1 from the original strain. This is confirmed by the literature data on the appearance of Xyl 3 in the proteome of the mutant strain *T. reesei* PC-3-7 while the absence of this enzyme synthesis in the original strain *T. reesei* QM9414.^12^ In addition, the proportion of β-xylosidase in the EP2 from *T. reesei*-Co-44 increased by 4.2% compared with the original strain, the proportion of xyloglucanase decreased from 2.1% to 0.2%, and the proportion of BGL remained almost unchanged.

**Figure 2.**
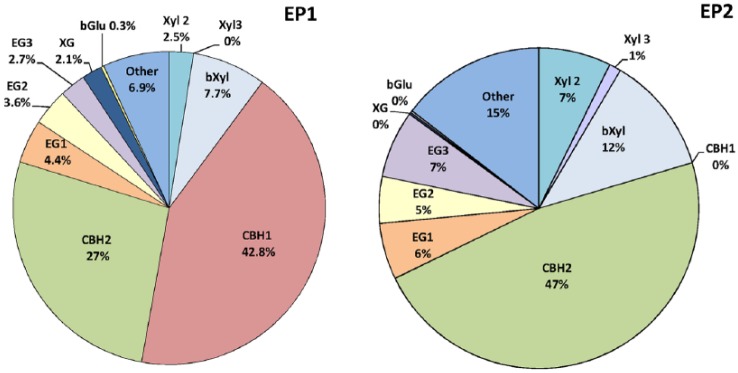
The content of individual carbohydrases in preparations based on the original strain (EP1) and cobalt mutant *Trichoderma reesei* Со-44 (EP2). CBH indicates cellobiohydrolase; EG, endoglucanase; XYL, xylanase.

Thus, gamma mutagenesis resulted in significant increase in endodepolymerases content in *T. reesei* Co-44 carbohydrases complex, which is confirmed by the results of the component composition study of EP obtained from culture liquids of the original and mutant strains. The complete absence of CBH I in the mutant strain enzyme complex can be considered as the most significant change that resulted from *T. reesei* gamma irradiation. An increase in the proportion of Xyl and EGs by 6% and 6.5%, respectively, also occurred in mutant strain.
